# P-861. CTX-M-15-producing *H*30 ST131 *Escherichia coli* contributes to carbapenem use for *E. coli* bloodstream infections in the United States

**DOI:** 10.1093/ofid/ofae631.1053

**Published:** 2025-01-29

**Authors:** Natalie A Mackow, Wanying Shao, Lizhao Ge, Lauren Komarow, Angelique E Boutzoukas, Jianping Jiang, Liang Chen, Erica Herc, Yohei Doi, Cesar A Arias, Owen Albin, Elie Saade, Loren G Miller, Jesse T Jacob, Michael J Satlin, Martin Krsak, W Charles Huskins, Sorabh Dhar, Samuel A Shelburne, Carol Hill, Kerryl Greenwood-Quaintance, Suzannah Schmidt-Malan, Robin Patel, Vance G Fowler, Pranita Tamma, Barry N Kreiswirth, David van Duin

**Affiliations:** University of North Carolina Chapel Hill, Chapel Hill, North Carolina; George Washington University, Rockville, Maryland; George Washington University, Rockville, Maryland; George Washington University, Rockville, Maryland; Duke University School of Medicine, Durham, North Carolina; Center for Discovery and Innovation, Hackensack Meridian Health, Nutley, New Jersey; SUNY-Buffalo, Buffalo, New York; Henry Ford Hospital, Detroit, Michigan; University of Pittsburgh, Toyoake, Aichi, Japan; Houston Methodist and Weill Cornell Medical College, Houston, TX; University of Michigan Medical School, Ann Arbor, MI; Case Western Reserve University, Cleveland, OH; Lundquist Institute at Harbor-UCLA Medical Center, Torrance, California; Emory University School of Medicine, Atlanta, GA; Weill Cornell Medicine, New York, NY; University of Colorado School of Medicine, CO; Mayo Clinic, Rochester, MN; Wayne State University/Detroit Medical Center, John Dingell VAMC, Detroit, Michigan; MD Anderson-University of Texas, Houston,, Texas; Duke Clinical Research Institute, Durham, North Carolina; Mayo Clinic, Rochester, MN; Mayo Clinic, Rochester, MN; Mayo Clinic, Rochester, MN; Duke University Medical Center, Durham, NC; Johns Hopkins School of Medicine, Baltimore, MD; Center for Discovery and Innovation, Hakensack Meridian Health, Nutley, New Jersey; University of North Carolina at Chapel Hill, Chapel Hill, NC

## Abstract

**Background:**

Drug-resistant *E. coli* is a leading cause of antimicrobial resistance-associated deaths globally. Specifically, resistance to ceftriaxone (CRO-R) is increasing in *E. coli*. High-risk clonal group ST131 and its pandemic *H*30 subclone are of high concern yet studies characterizing these infections are limited. We evaluated baseline characteristics and clinical outcomes associated with *H*30 ST131, non-*H*30 ST131 and non-ST131 *E. coli* bloodstream infections (BSI).

**Figure:**
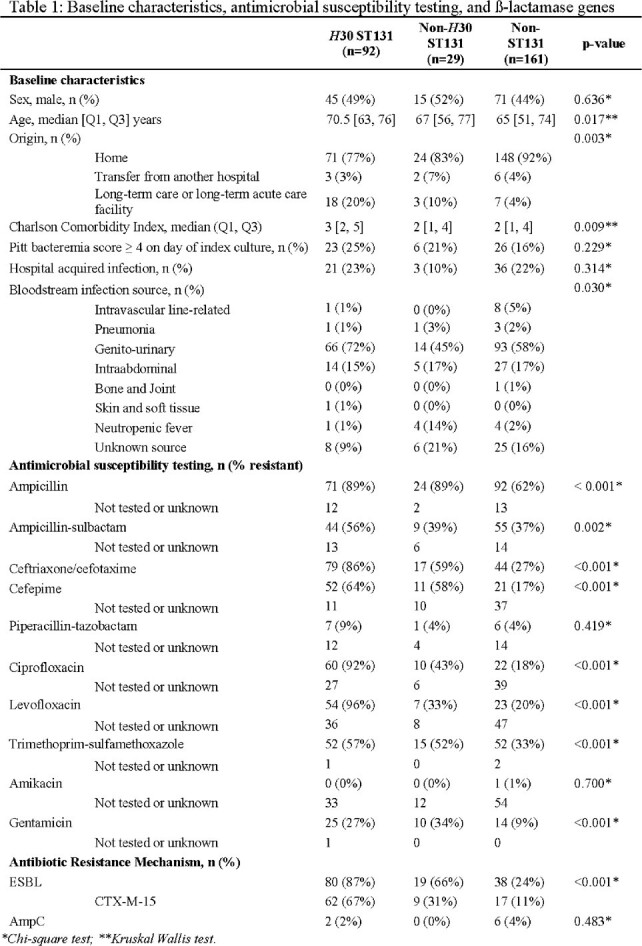

**Methods:**

Patients with monomicrobial carbapenem-susceptible *E. coli* BSI that were matched 1:1 by study site (CRO-R and CRO-susceptible community-acquired and hospital onset cases) were prospectively enrolled from 14 United States hospitals between November 12, 2020 to April 28, 2021 in the multicenter Study of Highly Resistant *E. coli* (SHREC). Isolates underwent whole genome sequencing. The primary outcome was a 30-day Desirability of Outcome Ranking (DOOR) after index culture including clinical response to treatment and all-cause mortality.

**Figure:**
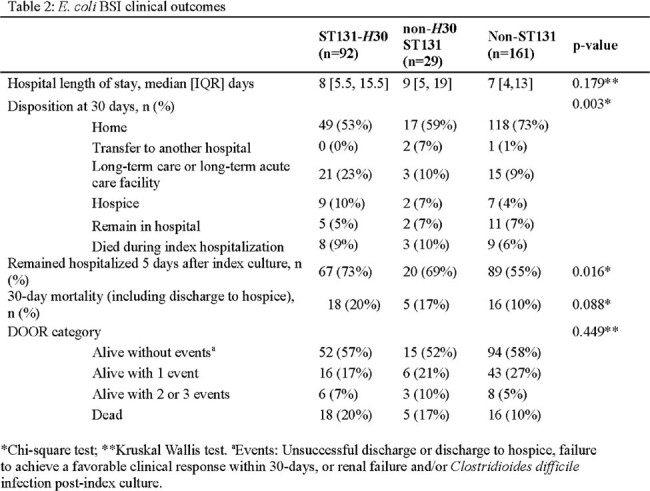

**Results:**

There were 92 (33%) *H*30 ST131, 29 (10%) non-*H*30 ST131, and 161 (57%) non-ST131 isolates in 282 *E. coli* BSI (Table 1). Most ceftriaxone resistance was conferred by CTX-M-15 produced by *H*30 ST131 isolates (Figure 1, Table 1). *H*30 ST131 BSI patients were older (median age [IQR] 70.5 [63,76] vs. 67 [56,77] vs 65 [51,74] years, p = 0.017), had higher Charlson comorbidity indices (3 [2,5] vs. 2 [1,4] vs. 2 [1,4], p=0.009), and were more often admitted from long-term care facilities (18/92 [20%] vs. 3/29 [10%] vs. 7/161 [4%], p = 0.003) compared to non-*H*30 ST131 and non-ST131 BSI patients. Among *H*30 ST131 isolates, high rates of antibiotic resistance were observed to cephalosporins and fluoroquinolones, resulting in significantly more carbapenem use compared with non-*H*30 ST131 and non-ST131 isolates (75/92 [82%] vs. 14/29 [48%] vs. 50/161 [31%], p < 0.001) (Figure 2). 30-day DOOR and hospital length of stay did not differ between groups (Table 2).

**Figure:**
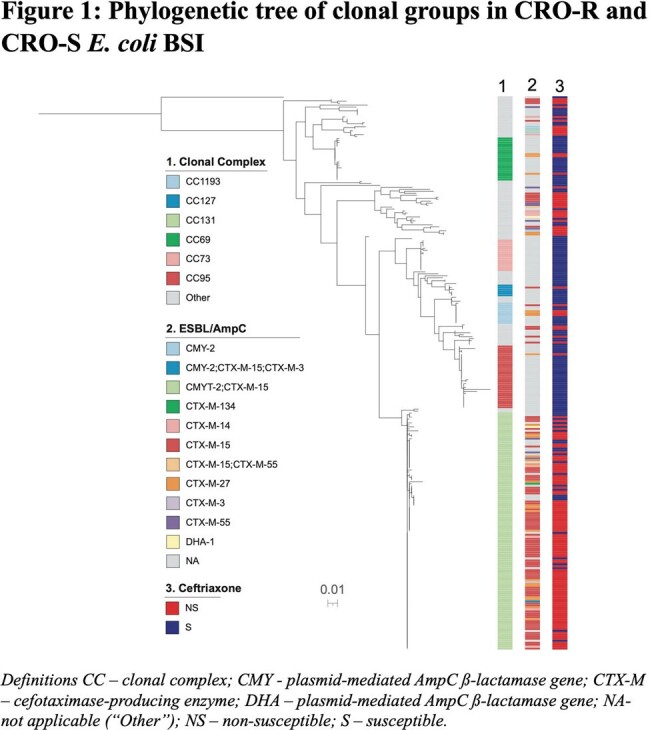

**Conclusion:**

Compared with non-*H*30 ST131 and non-ST131 *E. coli BSI, H*30 ST131 *E. coli* BSI have a unique epidemiology with more healthcare exposures, comorbidities and antibiotic resistance and are more likely to be treated with carbapenems, though no significant difference in clinical outcomes was observed.

**Figure:**
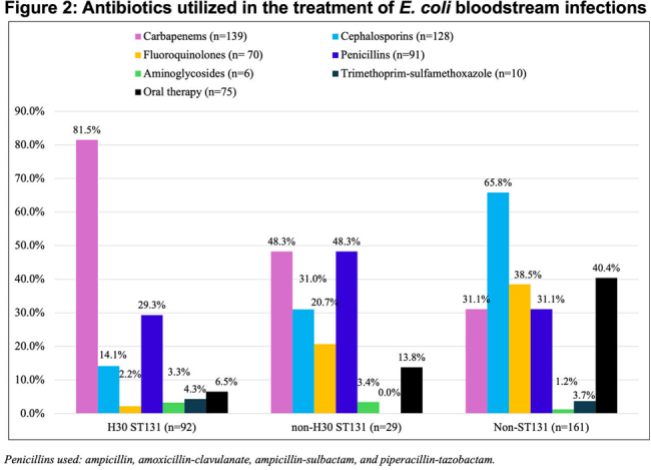

**Disclosures:**

**Yohei Doi, MD, PHD**, AbbVie: Honoraria|Entasis: Grant/Research Support|Gilead: Advisor/Consultant|GSK: Advisor/Consultant|Meiji Seika: Advisor/Consultant|Moderna: Advisor/Consultant|Pfizer: Advisor/Consultant|Shionogi: Advisor/Consultant|Shionogi: Honoraria **Elie Saade, MD, MPH, FIDSA**, Janssen Global Services: Advisor/Consultant|Janssen Global Services: Advisor/Consultant|Janssen Research and Development: Advisor/Consultant|Janssen Research and Development: Advisor/Consultant **Loren G. Miller, MD MPH**, Armata: Grant/Research Support|Contrafect: Grant/Research Support|GSK: Grant/Research Support|Merck: Grant/Research Support|Paratek: Grant/Research Support **Michael J. Satlin, MD**, AbbVie: DSMB participant|bioMerieux: Grant/Research Support|Merck: Grant/Research Support|Selux Diagnostics: Grant/Research Support|SNIPRBiome: Grant/Research Support **W. Charles Huskins, MD, MSc**, ADMA Biologics: Advisor/Consultant|Bristol Myers Squibb: Stocks/Bonds (Public Company)|Pfizer: Advisor/Consultant|Pfizer: Stocks/Bonds (Public Company)|Zimmer Biomet: Stocks/Bonds (Public Company) **Carol Hill, PhD**, Glaxo SmithKline: Retirement Health, Cash Balance Plan|Glaxo SmithKline: Stocks/Bonds (Public Company) **Robin Patel, MD**, a patent on Bordetella pertussis/parapertussis PCR issued, a patent on a device/method for sonication with royalties paid by Samsung to Mayo Clinic, a: See above|MicuRx Pharmaceuticals and BIOFIRE: Grant/Research Support|PhAST, Day Zero Diagnostics, Abbott Laboratories, Sysmex, DEEPULL DIAGNOSTICS, S.L., Netflix, Oxford Nanopore Technologies and CARB-X: Advisor/Consultant|Up-to-Date and the Infectious Diseases Board Review Course.: Honoraria **Vance G. Fowler, MD, MHS**, Affinergy: Advisor/Consultant|ArcBio: Stocks/Bonds (Private Company)|Armata: Advisor/Consultant|Astra Zeneca: Advisor/Consultant|Astra Zeneca: Grant/Research Support|Basilea: Advisor/Consultant|Basilea: Grant/Research Support|ContraFect: Advisor/Consultant|ContraFect: Grant/Research Support|Debiopharm: Advisor/Consultant|Destiny: Advisor/Consultant|EDE: Grant/Research Support|Genentech: Advisor/Consultant|Genentech: Grant/Research Support|GSK: Advisor/Consultant|Janssen: Advisor/Consultant|Karius: Grant/Research Support|MedImmune: Grant/Research Support|Merck: Grant/Research Support|sepsis diagnostics: Patent pending|UptoDate: Royalties|Valanbuio: Stocks/Bonds (Private Company)|Valanbuio: Stocks/Bonds (Private Company) **David van Duin, MD, PhD**, Merck: Advisor/Consultant|Merck: Grant/Research Support|Pfizer: Advisor/Consultant|Qpex: Advisor/Consultant|Roche: Advisor/Consultant|Shionogi: Advisor/Consultant|Shionogi: Grant/Research Support

